# Functional Structure of Spontaneous Sleep Slow Oscillation Activity in Humans

**DOI:** 10.1371/journal.pone.0007601

**Published:** 2009-10-26

**Authors:** Danilo Menicucci, Andrea Piarulli, Ursula Debarnot, Paola d'Ascanio, Alberto Landi, Angelo Gemignani

**Affiliations:** 1 Institute of Clinical Physiology, CNR, Pisa, Italy; 2 EXTREME Centre, Scuola Superiore Sant'Anna, Pisa, Italy; 3 Centre de Recherche et d'Innovation sur le Sport, Université Claude Bernard Lyon I, Lyon, France; 4 Department of Physiological Sciences, University of Pisa, Pisa, Italy; 5 Department of Electrical Systems and Automation, University of Pisa, Pisa, Italy; University of Alberta, Canada

## Abstract

**Background:**

During non-rapid eye movement (NREM) sleep synchronous neural oscillations between neural silence (down state) and neural activity (up state) occur. Sleep Slow Oscillations (SSOs) events are their EEG correlates. Each event has an origin site and propagates sweeping the scalp. While recent findings suggest a SSO key role in memory consolidation processes, the structure and the propagation of individual SSO events, as well as their modulation by sleep stages and cortical areas have not been well characterized so far.

**Methodology/Principal Findings:**

We detected SSO events in EEG recordings and we defined and measured a set of features corresponding to both wave shapes and event propagations. We found that a typical SSO shape has a transition to down state, which is steeper than the following transition from down to up state. We show that during SWS SSOs are larger and more locally synchronized, but less likely to propagate across the cortex, compared to NREM stage 2. Also, the detection number of SSOs as well as their amplitudes and slopes, are greatest in the frontal regions. Although derived from a small sample, this characterization provides a preliminary reference about SSO activity in healthy subjects for 32-channel sleep recordings.

**Conclusions/Significance:**

This work gives a quantitative picture of spontaneous SSO activity during NREM sleep: we unveil how SSO features are modulated by sleep stage, site of origin and detection location of the waves. Our measures on SSOs shape indicate that, as in animal models, onsets of silent states are more synchronized than those of neural firing. The differences between sleep stages could be related to the reduction of arousal system activity and to the breakdown of functional connectivity. The frontal SSO prevalence could be related to a greater homeostatic need of the heteromodal association cortices.

## Introduction

Sleep Slow Oscillations (SSOs) have been recently revealed and are increasingly attracting the attention of neurophysiologists as EEG characteristic signals during sleep [1]–[4]. In particular, it has been shown that individual events of SSO emerge from the background activity of the deepest stages of NREM sleep and spread over large areas of cortex [5]. This rhythmic activity, which originates in the cortex [6] and reverberates in subcortical structures [7], [8], was detected during sleep in humans through electroencephalographical (EEG) and magnetoencephalographical recordings [2], [3], [5], [9], and functional magnetic resonance imaging scans [10].

Although the mechanisms underlying this widespread synchronization and its propagation across cortical networks remain unclear, electrophysiological studies in animal models have revealed that during SSOs the membrane potential of cortical neurons shows a switching behavior: it oscillates between a state of hyperpolarization (down state) and a state of wake-like depolarization (up state), both lasting several hundreds of ms [1]. This behavior represents the fundamental cellular phenomenon underlying neural activity in slow wave sleep (SWS; NREM sleep stages 3 and 4) [6].

During the spontaneous activity of SWS, each SSO behaves as an event: it originates from a definite location, usually located in the anterior cortical regions, and propagates to posterior cortical areas as a traveling wave sweeping the scalp at a typical speed of a few m/sec. All these properties are highly reproducible across nights and in different individuals [5]. From a physiological standpoint, SSOs seem to play a role in sleep homeostasis [11]–[13] and, in particular, in the consolidation of recent memory traces [14]–[17].

In this paper we generalize the detection criteria of Massimini *et al.* [5] (MDC): We still detect propagating SSO events looking for waves satisfying these detection criteria but we also introduce a likeness rule to complete the identification of the events. More specifically, for each SSO event, this rule identifies concurrent SSOs which are very similar in shape, compared to those detected by the MDC, although sub-threshold. We analyzed sleep recordings from a 32-channel EEG system. From this sparse electrode array configuration typical for clinical use, we achieved results consistent with previous surveys obtained by high-density EEG [5].

The aim of this work is the detailed characterization of morphological and propagation-related features of SSOs *per se* and as a function of sleep stages and main cortical areas. We recorded the EEG during the first sleep cycle in individuals not engaged in any systematic task: SSO activity was expected during the NREM sleep.

## Materials and Methods

### Participants and experimental protocol

Ten right-handed (Edinburgh Handedness Inventory, EHI) non-sleep-deprived male participants signed an informed consent according to the University of Pisa Ethical Committee guidelines. Inclusion criteria were: age between 18 and 30 years; not having taken any medications for at least 1 year; no personal or family history of sleep disorders and no medical, neurological or psychiatric disorders, as assessed by semi-structured interviews.

We selected participants who had the same daily activity—i.e., students at the University of Pisa (Italy), spending at least 6 hours in class and reading scientific books. After an adaptation night, all volunteers were allowed to sleep at their usual bedtime and EEG recordings were carried out during the first sleep episode of the night.

### EEG recordings and pre-processing

A 40-channel (32-ch for EEG, 8-ch for auxiliary signals) DC-coupled, 22 bits, monopolar amplifier (Nuamps, Neuroscan, Compumedics, El Paso, TX) was used for signal recordings. The bandwidth was DC - 262.5 Hz, the input range was from −130 mV to +130mV with a sensitivity of 61 nV (19x gain). Signals were acquired with a sampling rate of 1 KHz. Electrode impedance was below 5 KOhm. Scalp EEG signals were referenced to the FCz potential.

Signal treatment (from pre-processing to wave analyses) was implemented using Matlab (MathWorks, Natick, MA, USA). All maps were obtained using the EEGLAB Toolbox [18].

Offline re-referencing to the average potential of the two earlobes (A1 and A2) was done in order to obtain monopolar recordings with a balanced distribution of contact impedances over the scalp, in line with previous specifications [19]. Also, we detected movement artifacts by analyzing all EEG channels and finding synchronous sudden increases in signal amplitude. We classified EEG epochs with amplitudes exceeding the threshold of the 95th percentile of the signal amplitude distribution as movement or muscular artifacts. After confirmatory visual inspection, we discarded most of these EEG epochs.

Finally, we checked for temporary declines in signal quality (instability or loss of contact with the skull during recordings) on the basis of signal statistics and excluded the detected bad channels (EEGLAB Toolbox [18]). At the end of these preprocessing steps, all recordings showed more than 90% of artifact-free epochs. These epochs were scored according to Rechtschaffen and Kales [20] criteria: the scoring of sleep stages was visually performed on the EEG referenced to the mastoid (C3-A2; C4-A1).

In line with previous works on SSO [2], [21], the retained EEG signals were offline band-pass filtered using a Chebyshev II filter (fdatool, MATLAB, The Math Works Inc, Natick, MA). Filter parameters were set in order to obtain minimal amplitude attenuation and phase distortion; the filter was applied on signals in both forward and reverse directions to avoid time biases. For experimental reproducibility, the complete specification of the filter parameters is reported below: low-frequency stop = 0.1 Hz, low-frequency pass = 0.5 Hz, high-frequency pass = 4.0 Hz, high-frequency stop = 4.4 Hz. The filtering procedure produced no attenuation at pass-band, −60 dB at the low stop-band and −80 dB at the high stop-band.

**Figure 1:**
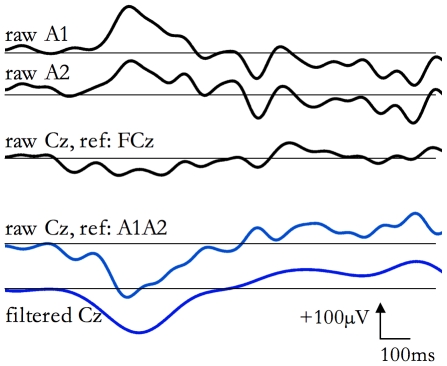
EEG re-referencing and filtering. The same SSO is shown. The upper three traces are the raw EEG (the channels Cz, A1 and A2, all referenced to Fcz) signals. In the middle, Cz signal is shown after re-referencing to the average between the two earlobe potentials (A1 and A2). At the bottom, the re-referred Cz trace, band pass filtered in the range 0.1–4.4 Hz is plotted.

The main steps of pre-processing are shown in Figure 1.

### Detection of SSO events

We scanned each EEG channel to detect SSOs according to the MDC [5]: a) two zero crossings separated by 0.3–1.0 s, with the first one having a negative slope; b) a negative peak between the two zero crossings with a voltage less than −80 µV; c) a negative peak to positive peak amplitude of at least 140 µV. It has been demonstrated that MDC are consistently matched both by sporadic K-complexes during stage 2 and by recurrent slow waves during the SWS [5]. From an operational point of view, since all detected waves matched the same criteria, irrespectively from the sleep stage of occurrence, we refer to all detected waves as sleep slow oscillations (SSOs).

**Figure 2:**
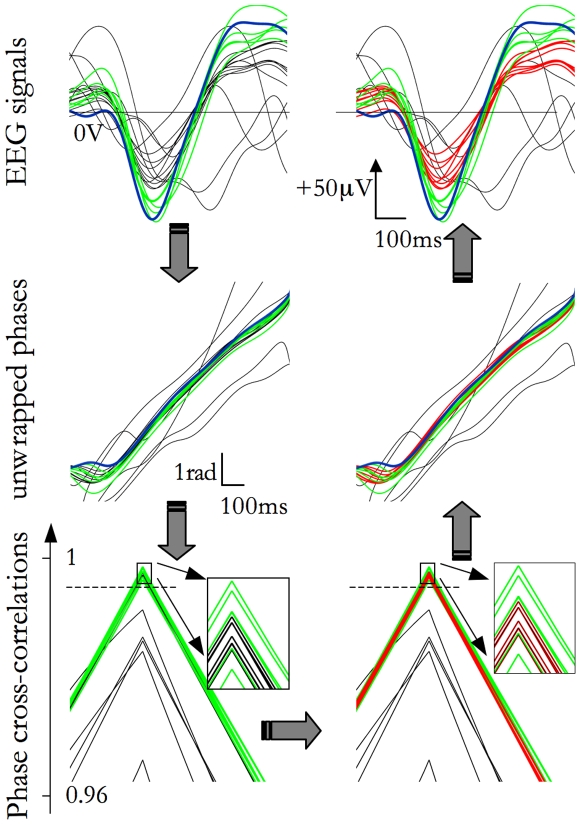
SSO event detection steps. Signals are scanned and SSOs are detected applying the Detection Criteria. A branch of concurrent SSOs, grouped in a event, are shown in the top left panel (blue and green lines, throughout all panels, the same color code has been used for each type of waves). The thickest blue trace identifies the ‘prototype’ of the event, namely the first SSO according to the temporal occurrence of its negative peak. In the middle panel on the left, instantaneous phases of the signals are plotted. In the bottom panel on the left, the cross-correlations functions between the prototype and the other traces are plotted. Applying the Likeness Constraint (dashed horizontal line), some sub-threshold SSOs are selected (red traces, in the bottom right panel). In fact, in the time-phase plane (middle panel on the right) well expressed and sub-threshold SSOs are superimposed. Also sub-threshold SSO signals are very similar to concurrent well-expressed SSOs (upper, right panel).

We used the negative peak latency to cluster concurrent SSOs detected on different channels: on the basis of published data [5], [21] and of a preliminary evaluation of our data, we considered SSOs to be concurrent if the maximal time lag among SSOs in the group was below 200 ms. As result, we considered each cluster as a single SSO event (Figure 2, blue and green traces in the upper, left panel).

### Sub-threshold SSO detection

For each detected event, we visually verified the presence of sub-threshold SSOs that were not detected in the previous step. In fact, the use of the MDC ensures the detection of well expressed, i.e. large, SSO but *a priori* prevents the detection of all the SSOs belonging to each event, like, for instance, in the case of ‘waxing and waning’ behavior during the SSO propagation. In addition, we observed that the strict application of the MDC to our 32-channel EEG data lead to SSO events propagating in spatially very limited cortical areas or, alternatively, jumping between non-contiguous areas. Both behaviors are in contrast with the SSO traveling nature [5].

To overcome this limitation, we introduced a likeness rule to include sub-threshold SSOs into detected events. This likeness rule is based on the fact that the SSO is sustained by alternating states of hyperpolarization and neural firing with a precise timing and, thus, tends to roughly maintain its shape during its propagation across the scalp. This means that SSOs belonging to each single event are very similar in shape.

We therefore calculated a within-event degree of likeness among detected SSOs to recover the undetected concurrent ones.

The proposed likeness rule works according to the following 4 steps.

1. For each event, the first SSO, sorted according to the temporal occurrence of its negative peak, is selected. We name it ‘prototype’ of the event (Figure 2, blue thick trace in the upper, left panel).
2. For each SSO event, the cross-correlation function C(m) between instantaneous phases (estimated by the Hilbert transform [22]) of the prototype signal and every other simultaneous EEG signal is estimated (Figure 2, middle, left panel). The C(m) function, estimated in a symmetrical time shift interval from −200 ms to +200 ms, always shows a cusp-like shape with a well-recognizable maximum max_m_(C). The maximum max_m_(C) occurs when the patterns of the two phases are maximally superimposed and its value provides an amplitude-independent estimation of the similarity between SSO waveforms (Figure 2, bottom, left panel).
3. For each recording, we calculated the distribution density of max_m_(C) derived from cross-correlation between EEG signals containing a concurrent detected SSO with its prototype. Notice that in this phase by the term ‘detected’ we mean signals passing the MDC. The 25th percentile of this distribution is assumed as the reference measure for the within-event SSO likeness (Likeness Constraint, dashed horizontal line in Figure 2, bottom, left panel).
4. The Likeness Constraint reference measure allows us:

a) to drop those events composed only by SSOs that are not very similar to their prototype -i.e., with all max_m_(C) values smaller than the Likeness Constraint;

b) to recover concurrent SSOs, namely waves more similar to their prototype than the Likeness Constraint (Figure 2, red traces in the right panels). Events are thus completed with new channels containing sub-threshold SSOs.

### Event characterization

In this section event characterization is described, first focusing on its propagation and then on its wave morphology. In both cases, we describe the set of measured features providing their physiological interpretation.

To characterize the propagation across the scalp of each SSO event, we estimated three features. 1) The *extent of propagation*, corresponding to the number of detected SSOs in the event. 2) The *extent of propagation in the area* – i.e., considering 4 main scalp areas, the number of detections per area normalized to the number of electrodes in the same area. The four main areas were defined as follows: frontal (Fp1, Fp2, F3, F4, Fz, F8, F7), central (FC3, FC4, C3, Cz, C4, CP3, CPz, CP4), temporal (FT7, FT8, T3, T4, TP7, TP8, T5, T6) and posterior (P3, Pz, P4, PO1, PO2, O1, Oz, O2). 3) The *speed of propagation*. The event speed was estimated resting on the assumption of the simplest propagation model: SSOs radially propagate from their event origin location, forming circular fronts like a stone thrown in a pond. The *event origin location*, *i.e.* the location from which the propagating SSO originates was detected searching for the channel containing the first occurring negative peak. Also the propagation delays were estimated by the latency between the negative peaks (precisely detectable since usually sharp and not crowned by oscillations in the spindle frequency range [5]).

The speed estimate was performed by calculating the linear regression between the distances from the event origin to each location of the electrode where an SSO was detected and the corresponding delays. The slope of the obtained linear interpolant corresponds to the estimated *speed of propagation*.

In summary, each event was characterized with three features: the *extent of propagation*, the *extent of propagation in the area*, and the *speed of propagation*. The *extent of propagation* is a measure of how much each SSO event spreads across the whole cortex, whereas the *extent of propagation in the area* indicates how each event spreads in each area. *Speed of propagation* gives information about the level of entrainment between neural patches, and hence between cortical regions [23].

**Figure 3:**
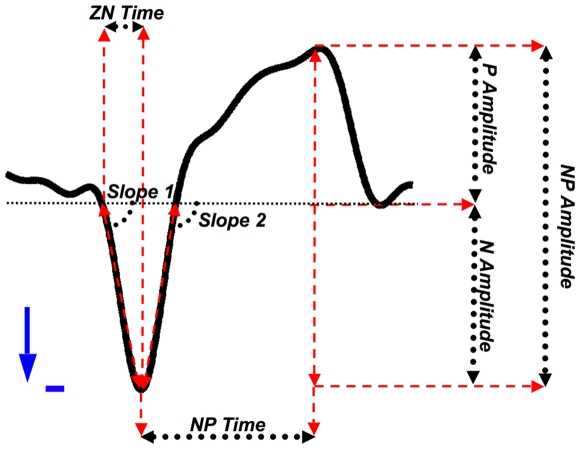
Graphical definition of the extracted morphological features. Each detected wave was characterized by means of seven features: three amplitude measures (the negative peak to positive peak amplitude - NP amplitude -, the negative peak amplitude - N amplitude - and the positive peak amplitude - P amplitude), two steepness measures (the absolute value of the slopes of the signal between the first zero crossing and the negative peak - slope 1 - and between the negative peak and the second zero-crossing - slope 2), and two different estimation of temporal width (the negative peak to positive peak time - NP time - and the first zero crossing to negative peak time interval - ZN time).

We next characterized the wave morphology of all the detected SSOs by 7 features (Figure 3). The feature set was composed of three amplitude measures (the negative peak to positive peak amplitude - *NP amplitude* -, the negative peak amplitude - *N amplitude* - and the positive peak amplitude - *P amplitude*), two steepness measures (the absolute value of the slopes of the signal between the first zero crossing and the negative peak - *slope 1* - and between the negative peak and the second zero-crossing - *slope 2*), and two different estimation of temporal width (the negative peak to positive peak time - *NP time* - and the first zero crossing to negative peak time interval - *ZN time*). Everywhere in the article, *italic* text identifies features names.

As far as the physiological meaning of morphological features is concerned, although several electrophysiological properties contribute to SSO shape, the SSO negative peak in the earlobe-referenced human sleep EEG is likely to mark the beginning of the transition to the depolarizing phase (*up* state) of the intracellular slow oscillation [1], [3], [24], [25]. Therefore, amplitude measures (*NP amplitude*, *N amplitude*, *P amplitude*) are mainly related to membrane potential amplitudes and to the size of the local neuronal pool showing a synchronized SSO behavior. Slope measures could be inherent to synchronization of the underlying neuronal pool during the transition towards the *down* state (*slope 1*) and during the transition between down and up states (*slope 2*) [26]. Consequently, *NP time* mainly reflects the duration of the complete transition between down and up states of the entire neuronal pool, whereas *ZN time* could be related to the duration of the down state, and therefore depends on mechanisms that are responsible for initiating and maintaining the hyperpolarization phase [27], [28].

### Statistical procedures

This section describes the statistical procedures for the event characterization with respect to sleep stages and main cortical areas. As far as cortical areas dependencies are concerned, we consider both the origin location of the event and the detection location of all SSOs belonging to each event. Dependencies were studied by using three main factors (everywhere in the article, uppercase initials identify these factors): Sleep Stage (NREM Stage 2 versus SWS), Origin Location (4 main areas) and Detection Location (4 main areas). The four main areas were the same used in the previous section to define the *extent of propagation per area*. For each feature earlier described we performed a statistical procedure aimed at unveiling the effect of the factors of interest adopting two approaches. For the features defined only per event, namely the *extent of propagation* and the *speed of propagation*, ANOVAs were performed with three between-subject factors: Subject (10 volunteers), Sleep Stage and Origin Location.

For the features defined per each SSO or per area, namely the morphological features and the *extent of propagation in the area*, ANOVAs were performed with the same three between-subject factors plus a within-subject factor (4 areas), that is the Detection Location factor.

ANOVA analysis studies if the feature marginal means of each factor level are statistically different one from another. As example, a significant effect for Origin Location factor means that the marginal mean of at least one area is different from the others. To derive which levels were significantly different from the others, pairwise comparisons (contrast analyses) were performed.

Moreover, for each feature, between-factors allow the recognition of effects between events whereas the within-factor studied effects among areas. It is worth noting that all between-subjects effects pertain to average values of each event. As consequence, no effect can depend on experimental differences in electrode location.

The results obtained from ANOVAs have been summarized in Tables and Figures. Tables aim focusing on significance of effects whereas Figures graphically report, for the features exhibiting a significant effect, contrasts among levels of the factor.

In this manner, large Tables of pairwise comparisons were not introduced; on the contrary, significant differences between factor levels have been depicted by a grayscale in simplified head maps. Each step in the grayscale indicates a significant difference (p<0.01) between contiguous tones. The adopted grayscale is ordinal, namely lighter tones correspond to higher relative values of the feature.

In addition, in each figure beside each grayscale head map the corresponding grand-average map has been depicted.

## Results

We performed EEG recordings in ten non-sleep-deprived individuals (males, age 20–30 years) during the first sleep cycle (session duration, 1.5–2 h). All individuals showed a physiological sleep EEG pattern, reaching the deepest stages of NREM sleep (stages 3 and 4, or SWS), with K complexes, spindles and delta (large waves in the 1–4 Hz frequency range) waves [20]. The sleep staging [20] allows the recognition of each first cycle. On average, the time spent in each stage (mean ± s.e.m.) expressed in percentage of recording time) was as the following: wakefulness-NREM stage 1, 25 ± 4%; stage 2, 33 ± 4%; and stages 3 and 4 (SWS), 35 ± 4%; REM sleep, 7 ± 1%.

The results of this work yield a quantitative description of the structure (both in term of shape and distributions of waves over the scalp) and of the propagation of spontaneous SSO events. In addition of verifying previous findings on SSO activity, we gave a detailed description of SSO characteristics and we statistically studied the possible effects on SSO features of factors such as SSO Detection Location, event Origin Location and Sleep Stage.

From each recorded session, the analyses were focused on both NREM Sleep Stage 2 and SWS. During the selected epochs, for each subject the detection algorithm identified several hundred of SSO events (400 ± 100; range: 131–905). The rate of occurrence of events during SWS (18±8 events/min) was significantly (p<0.001) higher than that observed during NREM sleep stage 2 (3.5±1.2 events/min).

**Figure 4:**
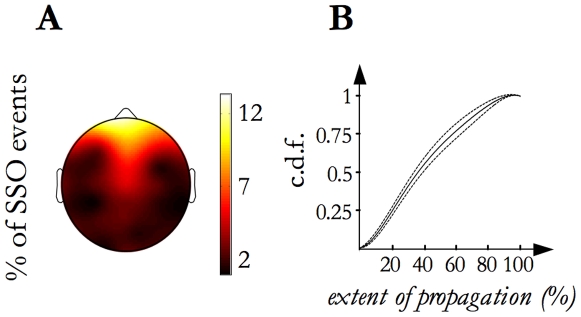
Origin location map and *Extent of propagation* distribution. A) The grand mean of the origin density map calculated from the individual maps is shown. It illustrates the frequency (reported as percentage) of SSO event origin for each electrode site. B) The thickest line represents the average (among subjects) cumulative distribution function (c.d.f.) of the *extent of propagation* and the two thinner lines correspond to the inter-subject confidence interval (p = 0.95).

We first studied the SSO event propagation across the scalp. We confirmed that each SSO event originates from a definite locus from which it spreads across the scalp. Figure 4, panel A shows the topographic map of *event origins*: 54% of events originated in the frontal area, 15% in the central, 17% in the temporal and 14% in the posterior. In particular, each of the prefrontal electrodes was the origin site of about 10% of events. Splitting per stage (stage 2 versus SWS), no differences (t- test per electrode, p<0.05) between maps emerged.

Next, the traveling behavior of SSO events was verified: we replicated that SSO events sweep the scalp with the most common pattern of propagation from frontal to posterior areas of the cortex at an average speed of 5±1 m/s (median, 3.4 m/s, with an inter-quartile range from 2.1 to 5.6 m/s). Also, the possible dependency of *speed of propagation* on between-subjects factors was considered. To this aim, ANOVA (three between-factors, that is Subject × Sleep Stage × Origin Location), as described in the Statistical Procedure subsection, was performed: no factors seem to systematically alter the *speed of propagation*.

We also measured the event *extent of propagation* as the percentage of EEG electrodes involved in each event. The cumulative distribution of the *extent of propagation* is shown in Figure 4, panel B: in line with previous findings derived from high density EEG recordings of spontaneous SSO activity [5], it ranges from 6% to 100% of the electrodes (namely, from 2 to 31 electrodes) with a median value of 39% (12 detections).

**Figure 5:**
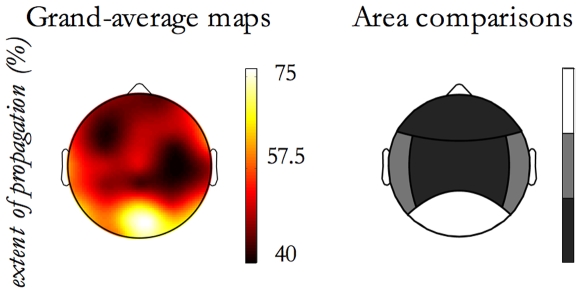
*Extent of propagation* versus Origin Location. For the *extent of propagation*, contrasts between Origin Location areas have been done. On the left, the *extent of propagation* grand-average map is depicted. In order to obtain a map related to the Origin Location effect, events originating from each origin site (electrode) have been selected and the grand-mean of extent of propagation per electrode has been derived. On the right, the contrast results are graphically reported by a grayscale code. Each step in the grayscale indicates a significant difference (p<0.01) between contiguous tones. The grayscale is ordinal, namely lighter tones correspond to higher extent of propagation values.

The *extent of propagation* was statistically studied as function of factors. ANOVA (design previously described) showed that events detected in NREM sleep stage 2 compared to SWS have a significantly higher *extent of propagation* (p<0.001). In addition, as shown in Figure 5, studying the Origin Location effect on the *extent of propagation*, it emerged that SSOs originating from the posterior area spread over a wider scalp surface than those originating from other areas, in particular the frontal ones (p<0.001).

**Figure 6:**
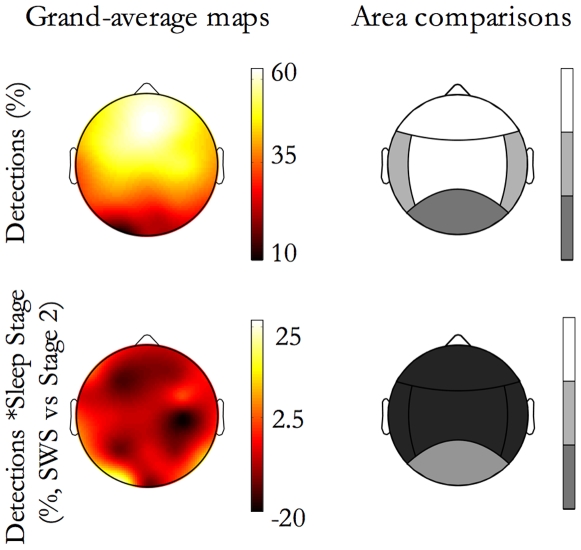
SSO detection location. The e*xtent of propagation in the area* is shown as function of Detection Location and Sleep Stage. On the left of the first row, the map of detections (% of events affecting each electrode) is depicted; on the right the corresponding contrasts between areas for the E*xtent of propagation in the area* are shown by a grayscale code. Each step in the grayscale indicates a significant difference (p<0.01) between contiguous tones. The grayscale is ordinal, namely lighter tones correspond to higher extent of propagation values. On the left of the second row, the difference between the detections in each stage (for each electrode, percentage of events in SWS minus same in stage 2) is depicted; on the right the corresponding contrasts between stages for the e*xtent of propagation in the area* are shown. The *extent of propagation in the area* is greater in NREM Sleep Stage 2 than SWS, except for SSOs detected in the posterior area. On the right column, a grayscale code indicates for each detection area if feature values are significant greater during SWS (white), or during sleep stage 2 (black) while grey indicates no difference between stages.

Beside the global *extent of propagation*, the distribution of detections per event over the scalp was also considered, also confirming previous findings [5]. The probability of detecting a slow oscillation during an event varies among electrodes (Figure 6, grand average map in the first row, on the left): on average, electrodes on the fronto-central regions are more likely involved in an event (single electrodes had a probability ranging from 50% to 70% of detecting an SSO during an event), whereas electrodes overlying the temporal and occipital cortices are only occasionally swept by spreading events (single-electrode probabilities from 7% to 35%).

We then studied the factor effects on the spatial distribution of detections by means of the *extent of propagation in the area*. ANOVA (General Linear Model repeated measures ANOVAs with three between-factors, that is Subject × Sleep Stage × Origin Location, and a within-factor, that is the Detection Location) yielded that frontal and central areas are those with the greatest *extent of propagation in the area*, while the posterior area is that with the lowest one (Figure 6, first row, on the right). Also, a sleep stage effect emerged: the *extent of propagation in the area* was greater in NREM sleep stage 2 than in SWS, with the exception of SSOs detected in the posterior area (Figure 6, second row).

**Figure 7:**
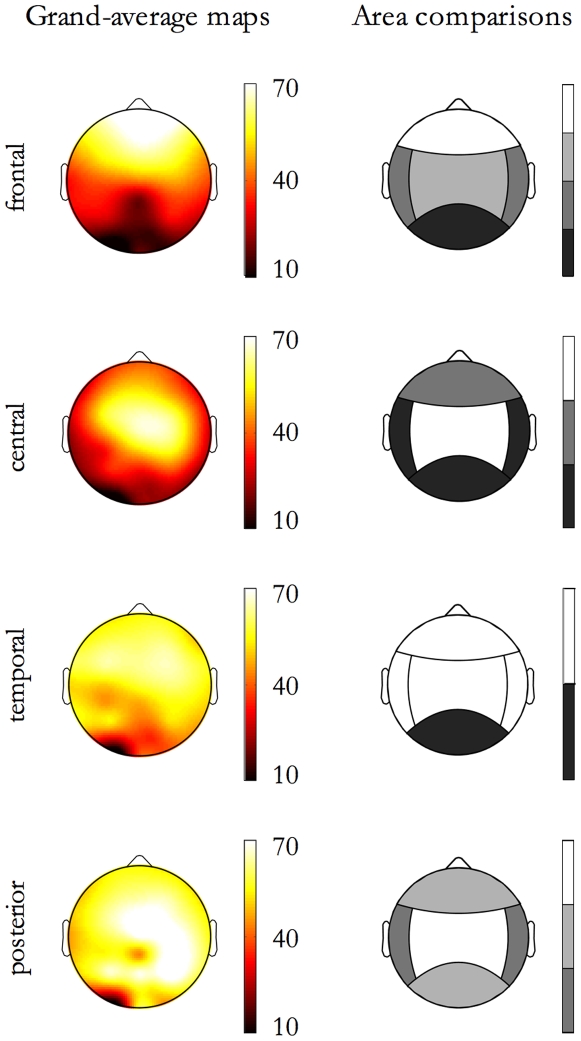
E*xtent of propagation in the area* as function of Origin Location. On each row, map related to events originating in an area are shown. On the left, the maps of detections (% of events affecting each electrode) are depicted; on the right the corresponding contrasts between areas for the E*xtent of propagation in the area* are shown.

As shown in Figure 7, *extent of propagation in the area* is dependent from the Origin Location. SSO events with frontal origin are minimally detected in the posterior area; those with a central origin preferentially spread toward the frontal area than in both temporal and posterior areas; those with a temporal origin do not spread in the posterior area; finally, those with posterior origin propagated preferentially in the central area.

**Figure 8:**
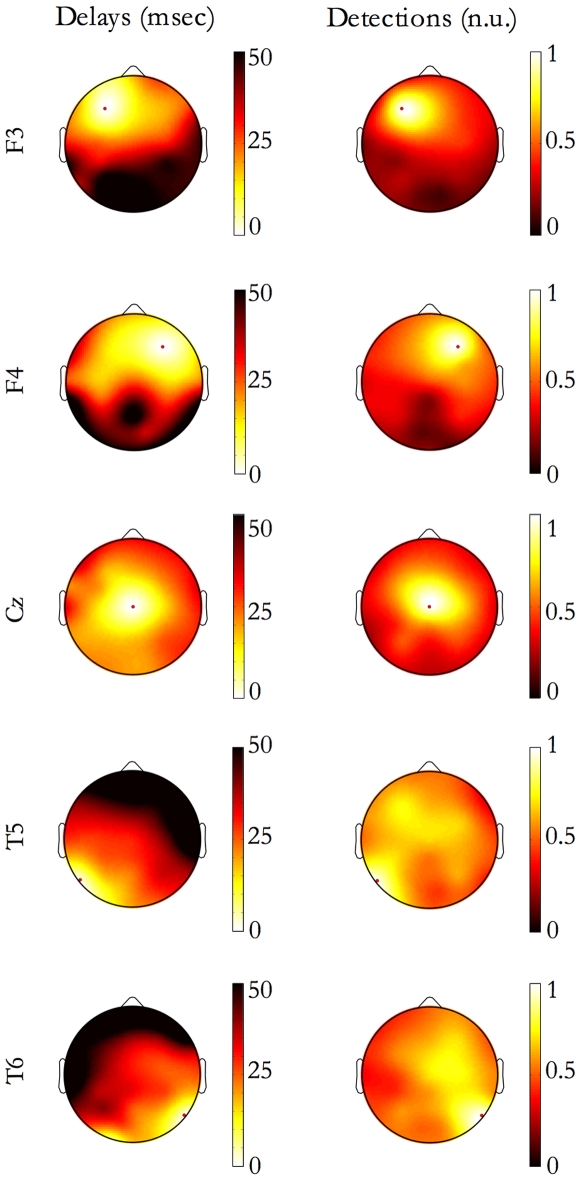
Continuity of event propagation. For events originating in 5 exemplary origin sites (F3, F4, Cz, T5, T6), panels depict the grand-average maps of delays (left column) and the grand-average maps of detections. The number of detections at each electrode site was normalized to the number of events originating in the red-dot marked site (normalized units, n.u.). The hemispheric cortical separation does not seem to influence the radial propagation of SSO events.

We investigated whether the hemispheric separation affects the SSO propagation. For all origin locations, at least 75% of SSO events propagate to the contralateral hemisphere (averaging among electrodes, 91% of events are bilateral); in addition, most of the unilateral SSO events are local since, on average, they affect only 4 electrodes, instead of the general *extent of propagation* of 12 electrodes. Indeed, considering single sites (electrode locations) of origin, the corresponding maps of delays (Figure 8, left column) are qualitatively in line with the isotropic model of propagation earlier used for speed estimation, and the scalp midline do not affect the propagation. Also, the maps of detections (Figure 8, right column) do not show any barrier between hemispheres.

To study the directions of propagation as function of the origin site, we verified the null-hypothesis of an isotropic radial propagation, namely a propagation from the origin without a preferential direction. For geometrical reasons, this implies that for each origin site the mean direction of propagation should be oriented toward the head centre (Cz).

**Figure 9:**
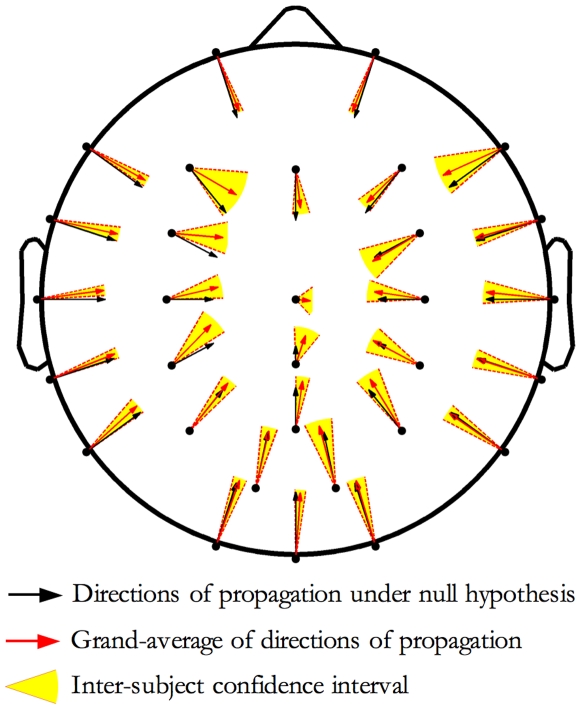
Directions of propagation. For each origin site, the black vector indicates the null hypothesis direction (isotropic radial propagation), while the red one indicates the estimated grand-mean direction and the yellow circular sector depicts the variability (95% confidence interval) between individuals. Data indicate a systematic tendency to drift toward frontal areas.

In Figure 9, for each origin site, the black vector indicates the null hypothesis direction (namely to the head centre) while the red one indicates the estimated grand-mean direction and the yellow circular sector between the two dashed black lines depicts the variability (95% confidence interval) between individuals. As illustrated in Figure 9, the mean directions of propagation point systematically, but not statistically significantly, to areas more frontal than the head vertex. In addition, splitting per stage (stage 2 versus SWS), no differences (t-test per electrode, p<0.05) between directions of propagation emerged.

Apart from their spatial occurrence over the scalp, the waveform characteristics of SSOs were studied measuring a set of morphological features (Figure 3, and for further details see ‘Events Characterization’ subsection in ‘Materials and Methods’): *NP amplitude*, *N amplitude*, *P amplitude*, *NP time*, *ZN time*, *slope 1*, *and slope 2*.

Table 1 summarizes the descriptive statistics of individual mean values of each event feature. It is worth noting that the *NP amplitude* is smaller than the threshold value used to detect SSO events by Massimini *et al.* [5]. The reason lies in the generalization of the MDC that enables the detection of SSO events, independently from the wave amplitude evolution during the propagation, even in the case of “waxing and waning” behavior. As a consequence of selecting individual sub-threshold SSOs, the average *NP amplitude* of the SSO events assumes lower values.

**Table 1:** Individual mean values of each SSO feature.

	*Mean ± Standard Error*	*Percentiles*		
Features		*25 th*	*50 th*	*75 th*
*NP Amplitude (mV)*	138 ± 7	109	128	155
*N Amplitude (mV)*	-86 ± 4	-104	-82	-63
*P Amplitude (mV)*	46 ± 4	23	41	62
*NP Time (ms)*	486 ± 21	382	453	555
*ZN Time (ms)*	271 ± 11	170	236	339
*slope 1 (mV/ms)*	-0.38 ± 0.01	-0.47	-0.36	-0.25
*slope 2 (mV/ms)*	0.34 ± 0.01	0.25	0.32	0.4

Group mean with standard error and quartile values are indicated.

Table 1 shows that the group average of *slope 1* is steeper than that of *slope 2*. Indeed, we observed that the within-subject average of *slope 1* was steeper than *slope 2* for all individuals. This allowed rejecting the hypothesis of same steepness for the two slopes (Wilcoxon ranksum test [29], p<0.05).

The possible dependency of the morphological features from sleep stage, detection and origin areas factors were evaluated by ANOVAs (General Linear Model repeated measures ANOVAs with three between-factors, that is Subject × Sleep Stage × Origin Location, and a within-factor, that is the Detection Location). We found a strongly significant Subject effect (p<0.001) for all features, and contrast analysis indicated that every individual was different from any other. We also found a significant Sleep Stage and Origin Location effect for some features (Table 2). Lastly, interaction between Sleep Stage and Origin Location was not significant for any feature.

**Table 2:** Between-events effect analysis of SSO features.

	Factors		
	Sleep Stage		Origin Location
	p-val	Level comparisons	p-val
Features	prob.		prob.
*NP Amplitude*	<0.001	SWS>S2	NS
*N Amplitude*	NS	–	NS
*P Amplitude*	<0.01	SWS>S2	NS
*NP Time*	<0.01	SWS>S2	<0.001
*ZN Time*	NS	–	NS
*slope 1*	NS	–	<0.01
*slope 2*	<0.01	S2>SWS	<0.05

The results corresponding to each feature have been organized in two main columns related to the Sleep Stage and Origin Location factors. In the first column, significance and direction of difference between SWS and NREM Sleep Stage 2 are shown; while in the second column, significance of Origin Location effect is reported.

Table 2 is organized into two main columns, corresponding to the Sleep Stage and Origin Location factors. Significance and direction of difference between SWS and NREM Sleep Stage 2 are shown in the first column; whereas the second column reports significance of Origin Location effect.

From the first column, it derived that SSOs during SWS are wider, both for waves amplitude and for the duration of the transition from the down state to the upstate (SSOs detected during SWS have greater *NP amplitude*, *N amplitude* and *NP time* than those during NREM stage 2).

**Figure 10:**
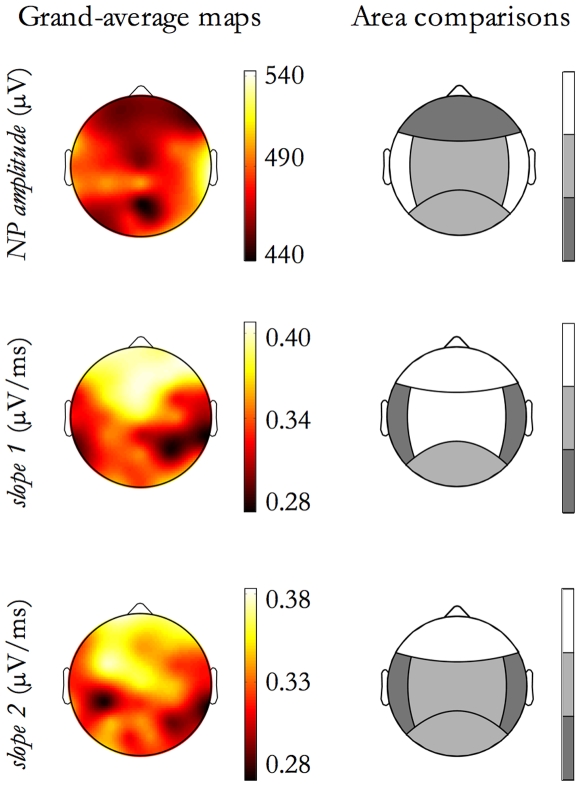
Morphological features as a function of Origin Location. For features exhibiting an Origin Location effect, as reported in Table 2, contrasts between Origin Location areas is shown. Each row corresponds to a feature. On the left column, the feature grand-average map is depicted. Since maps correspond to the Origin Location effect, events originating from each origin site (electrode) have been selected and the grand-mean, for each electrode, has been derived. On the right column the contrast results are graphically reported by a grayscale code. Each step in the grayscale indicates a significant difference (p<0.01) between contiguous tones. The adopted grayscale is ordinal, namely lighter tones correspond to higher relative values for the feature.

From the second column, it derived that the SSO slopes depend from the site of event origin. To identify which origin areas differ from the others, we performed contrast analyses among origin locations. For the morphological features having a significant Origin Location effect, Figure 10 shows both its topographical scalp map and the significance of differences among areas (each step in the grayscale indicates a significant difference -p<0.01- between contiguous levels).

Figure 10 indicates that *NP time* was shorter for SSO events originating in the frontal area than for those in the central and posterior areas; *slope 2* behaves in the opposite manner. Furthermore, *slope 1* was steep for SSO events with a fronto-central origin and gentle for SSO events with a temporal origin.

Moreover, results concerning the differences in SSO morphology as a function of Detection Location (the within factor), are summarized in Table 3. The two columns respectively correspond to the main event effect of Detection Location and to the interaction between Detection Location and Sleep Stage factors.

**Table 3:** Within-*event* effects analysis of SSO features.

	Factors	
	Detection Location	Detection Location * Sleep Stage
Features	p-val	p-val
*NP Amplitude*	0.001	0.01
*N Amplitude*	0.001	NS
*P Amplitude*	0.001	NS
*NP Time*	NS	NS
*ZN Time*	0.001	NS
*slope 1*	0.001	NS
*slope 2*	0.001	0.001

The results corresponding to each feature have been organized in two main columns related to the main Detection Location effect and to the interaction between Detection Location and Sleep Stage. In each column, the significance of each effect is shown.

**Figure 11:**
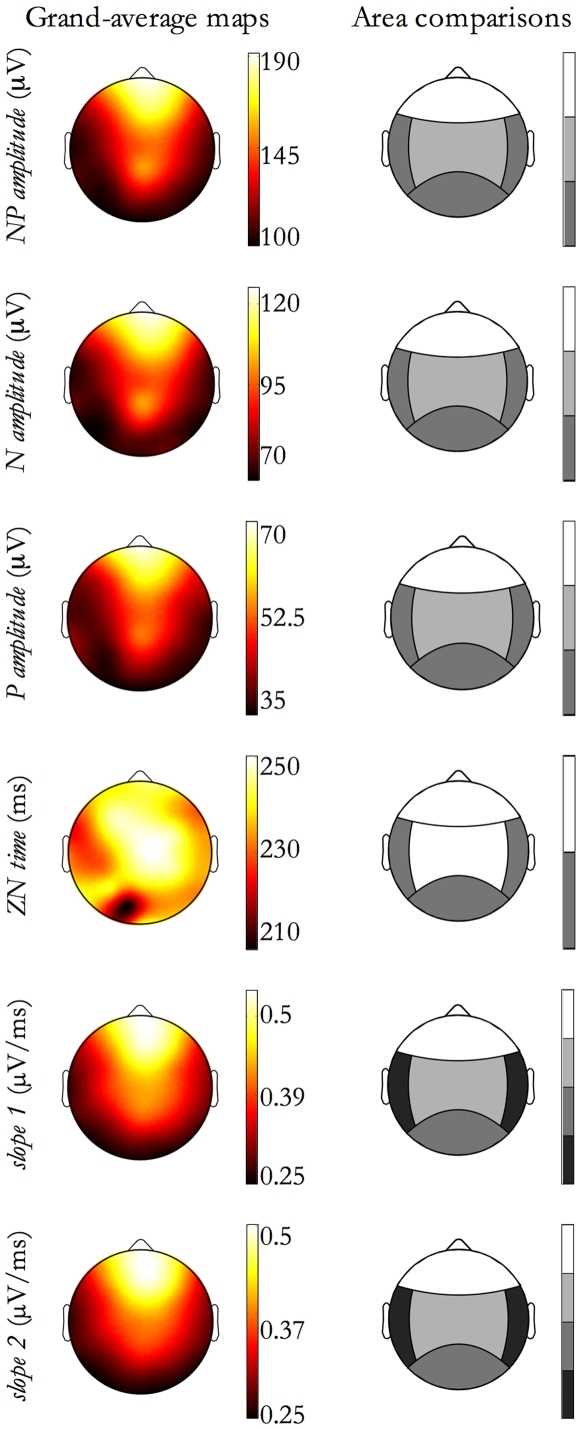
Morphological features as a function of Detection Location. For features exhibiting a Detection Location effect, as reported in Table 3, contrasts between Detection Location areas have been done. In the figure, each row corresponds to a feature. On the left column, the feature grand-average map is depicted. Since maps correspond to the Detection Location effect, SSOs detected in each electrode site have been selected and the grand-mean, for each electrode, has been derived. On the right column the contrast results are graphically reported by a grayscale code. Each step in the grayscale indicates a significant difference (p<0.01) between contiguous tones. The grayscale is ordinal, namely lighter tones correspond to higher relative values for the feature.

For the features showing a significant effect of Detection Location, contrasts analyses were performed. Figure 11, right column, shows, by the grayscale code, significant between-areas differences. In the left column of Figure 11, the corresponding grand-average maps are shown.

As shown in Table 3, first column and in Figure 11, the study of dependencies on the Detection Location factor showed that SSOs detected in frontal regions, independently from origin site, have the highest amplitude and duration, whereas SSOs detected in temporal and posterior areas have the lowest amplitude and duration.

**Figure 12:**
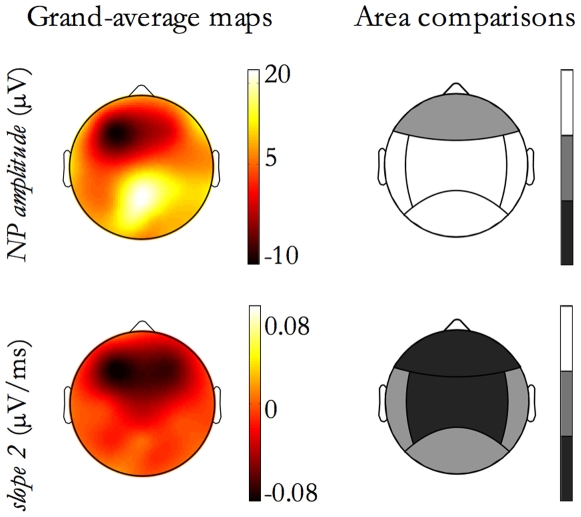
Morphological features as a function of Sleep Stage. For features exhibiting a significant interaction between Detection Location and Sleep Stage, as reported in Table 3, the figure shows, for each area, comparisons between sleep stages. In the figure, each row corresponds to a feature. On the left column, grand-average maps derived subtracting Sleep Stage 2 values from SWS values are shown. On the right column, a grayscale code indicates for each detection area if feature values are significant greater during SWS (white), or during sleep stage 2 (black) while grey indicates no difference between stages.

For the features with a significant interaction effect in Table 3, Figure 12 shows, on the right column, the grayscale code indicating for each detection area if feature values are significantly greater during SWS (area colored in white), or during NREM Sleep Stage 2 (black). A grey-colored area indicate no difference between stages in the area. Also, on the left column, the corresponding topographic map is shown: it is worth noting that these maps depict the differential behavior between stages and thus they were obtained subtracting NREM sleep stage 2 map from SWS map.

As shown in Table 3, second column and in Figure 12, the study of interaction between Detection Location and Sleep Stage produced some significant findings. *NP amplitude* and *slope 2* significantly changed their within-area mean value during SWS with respect to NREM sleep stage 2. In particular, *NP amplitude* of SSO detected in central, temporal and posterior areas was larger during SWS, while *slope 2* appeared steeper during NREM Sleep Stage 2 for SSOs detected in the frontal and central regions.

At variance, none of the morphological features showed significant interaction between Detection Location and Origin Location.

## Discussion

In this paper we statistically characterized the spontaneous SSO activity in non-sleep deprived volunteers . Participants had a physiological sleep EEG pattern, showing a higher rate of SSO events during SWS than during NREM stage 2. SSOs were detected all over the scalp but with a prevalence over the fronto-central areas and, on average, SSOs travel at a mean speed of 5 m/s, mainly originating in the frontal area and crossing the scalp to posterior regions. These findings constitute a replication of the original study of Massimini el al. [5] with the significant variation of obtaining confirmatory results from a sparse array of EEG electrodes instead of high-density EEG. We generalized the SSO detection algorithm used by Massimini *et al.* [5] proposing the likeness rule that allowed the selection of channels containing sub-threshold SSOs. At variance with *Riedner* et *al*. [21], we still detected only full-fledged SSO events. Namely, the events we detected complied the detection criteria of *Massimini* et *al*. [21] on at least one site of detection: this allows for the detection of SSOs irrespectively from the wave amplitude evolution during the event propagation. On the contrary, *Riedner* et *al*., dropped the amplitude criteria, maintaining only the temporal constraint: this approach on the one hand guarantees the detection of almost all the SSO events in EEG recordings, but on the other hand it may lead to the detection of events composed only by very low-amplitude waves (less than 50 µV), far from being correspondent to the paradigmatic SSO or, even more, to the paradigmatic K-complex.

In our opinion, the detection of sub-threshold SSOs successfully completes the identification of EEG patterns underpinning alternating silent and active neural states of the slow oscillation and, at the same time, yields a low number of false detections.

In this paper we adopted the perspective of considering both K-complexes and SSOs as a unitary phenomenon that occurs at rates increasing with sleep stages [5], [30], [31]. However, the underlying phenomenon may change with continuity with the depth of sleep, generating differences between waves occurring during NREM stage 2 and during SWS. The lack of up states recently identified in K-complexes of stage 2, compared to slow waves of SWS [31] could be interpreted within this perspective as well as the significant Sleep Stage effects that we identified.

The descriptive statistics shown in Table 1 may constitute a first reference for healthy subjects, with the limitations deriving from the small sample, the narrow age range and the prevalent daily activity performed by all individuals. The statistics of slopes suggested that *slope 1* is steeper than *slope 2*, and paired tests confirmed this observation. This finding is consistent with Volgushev *et al.* [32], who showed, by simultaneous multisite intracellular recordings in cats, a long-range synchronization in neocortical neurons during SWS. Their study demonstrated a precise synchronization both in down and up states; however, onsets of silent states were more synchronized than those of neural firing. Our data suggest that, also in humans, transitions to down states are more synchronous than transitions from down- to up states, within neural assemblies: thus the mechanisms underlying slow oscillations might though to be the same among mammals.

Aside from the descriptive statistics of SSO features, the main novel results of this work are the determination of effects on SSO event characteristics (both morphological and propagation-related) due to factors such as the sleep stage, the area of SSO detection, the event origin location.

The Sleep Stage effect on SSO activity identified a dichotomous behavior: during SWS the cortical network was more prone to locally induce SSOs, but less able to spread this behavior across the cortex, in comparison with NREM stage 2.

The increases of both amplitude measures and *slope 2* (i.e., the measure of synaptic synchronization strength within the underlying neuronal pool engaged in the SSO [21]) moving from NREM stage 2 to SWS seem to indicate that the cortical proneness to SSO increases as sleep deepens. This could be related to the decreased firing of neurons in the midbrain reticular formation and mesopontine cholinergic nuclei, which removes a steady excitatory drive from thalamocortical neurons and allows the membrane potential of cortical neurons to reach more hyperpolarized levels [30].

The decrease of the SSO-spread capability moving from NREM stage 2 to SWS could be related to the breakdown of the cortico-cortical connectivity in the transition from wakefulness to deep sleep. In fact, on the one hand it has been shown that during SWS the evoked TMS responses tend to be segregated in the stimulated area [33] and, on the other hand, acoustic-evoked K complexes are more distributed in the temporal and occipital cortex during NREM stage 2 than during SWS [34]. We suggest that also SSO events during SWS, like K-complexes, may inhibit the cortical response to external stimuli, favoring the deepening of sleep.

The study of both Origin and Detection Location effects on slope and amplitude measures indicate that the frontal area network is more able to produce SSOs than networks in the posterior and temporal areas. Also the directions of propagation are slightly biased toward frontal regions. Indeed, events originating in the posterior area are the most widespread, since they travel to the central and frontal areas that are the prominent propagators of the activity. On the other hand, frontal- and central-originating SSOs often halt their propagation when reaching the posterior area.

At present, the reasons why some areas seem to be more able to produce an SSO-like behavior than others are still unclear. It has been hypothesized that only certain brain areas possess the necessary dendritic geometry to produce such large scalp potentials [35]. It is also interesting to note that our data indicated that the topography of origins, the location of detections, the propagation directions and the slope/amplitude measures are all coherently biased toward the heteromodal association cortices. Increasing evidence suggest that during waking, higher-order cortical areas such as heteromodal association cortices undergo more plasticity compared to primary sensory cortices and show the greatest metabolic reduction throughout the sleep period [36]. In addition, using animal models, it has been described that increasing the metabolic rate of plastic brain structures during wakefulness, also the NREM delta activity in subsequent sleep increases [37]–[39]. Finally, the *synaptic homeostasis hypothesis* [11], [12] suggested that a sort of synaptic strength might undergo a homeostatic regulation related to plastic processes [21], [26].

We confirm that, on average, SSO events propagate orderly over the scalp. As far as fronto-posterior axis of propagation is concerned, it has been suggested [35] the existence of a mesial highway that may include the anterior and the posterior cingulate as well as the precuneus. Also the interhemispheric propagation didn't show any barrier at the midline, and the propagation of SSOs to contralateral sites is consistent with various pathways. Although a role of reticulothalamic or brainstem structures cannot be excluded [10], [40], [41], an involvement of the homotopic transcallosal fibers has been proposed. In fact, the corpus callosum is involved in EEG delta wave coherence between the two hemispheres [42] and, during the down state of SSOs, fast-rhythmic-bursting neurons could efficiently maintain the interhemispheric synchronization during all phases of SSO firing in response to callosal volleys [43].

As a conclusion, this work gives a quantitative picture of spontaneous SSO activity during NREM sleep. It focuses on shape and propagation of SSOs, while further studies could consider how SSOs modulate other rhythms such as spindle or faster activities. The same statistical approach could be applied on spectral measurements such as the modulation of power or phase locking phenomena as function of cortical areas and sleep stages.

Starting from the depicted SSO activity arrangement, any feature (its mean value or its distribution among the areas) could be liable to change as a consequence of a variety of tasks [14], [44] and we hypothesize that also sleep disorders could contribute to change specific SSO features. Further works will verify whether SSO shapes and dynamics might help comprehension and early detection of both local cortical deficits and pathological interactions in large-scale brain networks.
